# Sleep scoring made easy—Semi-automated sleep analysis software and manual rescoring tools for basic sleep research in mice

**DOI:** 10.1016/j.mex.2015.04.005

**Published:** 2015-04-24

**Authors:** M. Kreuzer, S. Polta, J. Gapp, C. Schuler, E.F. Kochs, T. Fenzl

**Affiliations:** a Department of Anesthesiology, Klinikum rechts der Isar, Technische Universität München, Munich, Germany; b Max Planck Institute of Psychiatry, Munich, Germany; c Institute of Pharmacy, Department of Pharmacology and Toxicology, University of Innsbruck, Innsbruck, Austria

**Keywords:** Semi-automated sleep scoring, Sleep scoring, Vigilance states, EEG, EMG

## Abstract

Studying sleep behavior in animal models demands clear separation of vigilance states. Pure manual scoring is time-consuming and commercial scoring software is costly. We present a LabVIEW-based, semi-automated scoring routine using recorded EEG and EMG signals. This scoring routine is

• designed to reliably assign the vigilance/sleep states wakefulness (WAKE), non-rapid eye movement sleep (NREMS) and rapid eye movement sleep (REMS) to defined EEG/EMG episodes.
• straightforward to use even for beginners in the field of sleep research.
• freely available upon request.

Chronic recordings from mice were used to design and evaluate the scoring routine consisting of an artifact-removal, a scoring- and a rescoring routine. The scoring routine processes EMG and different EEG frequency bands. Amplitude-based thresholds for EEG and EMG parameters trigger a decision tree assigning each EEG episode to a defined vigilance/sleep state automatically. Using the rescoring routine individual episodes or particular state transitions can be re-evaluated manually. High agreements between auto-scored and manual sleep scoring could be shown for experienced scorers and for beginners quickly and reliably. With small modifications to the software, it can be easily adapted for sleep analysis in other animal models.

## Graphical abstract

**Figure:**
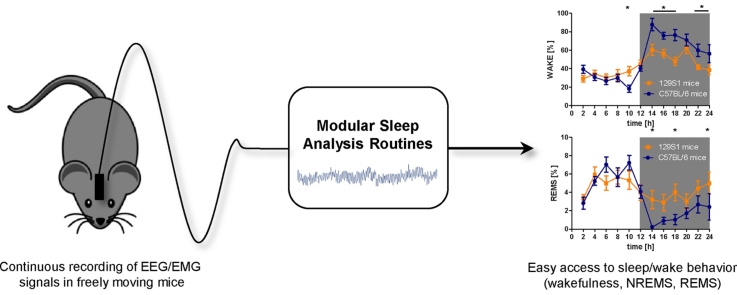

## Method details

The presented sleep scoring routine enables fast and easy semi-automated definitions of the vigilance/sleep states wakefulness (WAKE), non-REM sleep (NREMS) and REM sleep (REMS), especially for beginners in this field. The underlying algorithms applied in the present program are based on the vigilance/sleep state classification algorithm introduced by Louis et al. for rats, modified for mice by Fenzl and co-workers [6], [3].

The program was written in LabVIEW 8.5 (National Instruments, Austin, TX, USA). Starting the sleep scoring software a graphical user interface GUI will be opened and the user can choose between three executable programs: A manual ARTIFACT DETECTION routine, the SLEEP SCORING routine for semi-automated sleep scoring and a RESCORING routine to enable manual re-evaluation of distinct automatically scored EEG/EMG sequences.

In total, 14 mice were used for this study. All mice where kept in their home cages (26 cm × 26 cm with wood chips and nesting material) during the whole experiment under constant light/dark cycles (12/12 h; 220 lx in the light period) and constant temperature (24 °C ± 1 °C) within noise reduced recording chambers. Water and food was supplied ad libitum. All mice were allowed to recover from surgery for 14 days. Within that period all mice were also adapted to the laboratory light/dark cycles. After recovery, EEG and EMG recordings started 23 h a day for at least 3 consecutive days. The one hour gap (at the end of the dark period) after 23 h of recording and the beginning of the next 23 h recording was used for animal maintenance, if needed.

The animals were surgically prepared under isoflurane anesthesia (Univentor 410 anesthesia unit, agnthós, Sweden) within a stereotactic frame (Angle Two Leica Biosystems, USA). After surgical tolerance was reached, the animals’ eyes were covered with eye ointment to prevent desiccating and each animal received Meloxicam (0.5 mg/kg) to reduce postoperative pain. The analgesic was additionally given into the water bottle (0.5 mg/kg) for 8 days following surgery. EEG and EMG electrodes were composed of gold wire with ball shaped endings of approximately 100 μm diameter and were surgically implanted in all animals. The electrodes were soldered on socked boards (Typ 861-87-008, preci-dip, Switzerland) that were used to connect the recording cable [7], [4]. The recording cable consisted of a headstage amplifier (1x amp., npi electronics, Germany), connected to a commutator (SL-10, slip-ring commutator, Dragonfly, USA). The commutator was balanced on a swivel equipped with a counter weight (custom made, Streicher, Austria). This design facilitated weight-neutral, free movements of the animals in all three dimensions within the home cage. Signals of two EEG electrodes, a ground electrode and a differential electrode together with two EMG signals were fed into individual amplifiers (type DPA-2FL, npi electronics, Germany). The amplification factor of the EEG and EMG recording was set to 1000. Before analog to digital conversion, EEG signals and EMG signals were band pass filtered to the 0.1–100 Hz range. Digital sampling rate was 250 Hz (Power 1401-3 AD-board and SPIKE2, Cambridge Electronic Design, UK). The EMG signals were additionally band pass-filtered online (40–90 Hz). All signals were stored for offline analysis.

All experiments were conducted under the guidelines of national and international animal welfare protocols and were approved by the Bundesministerium für Wissenschaft, Forschung und Wirtschaft, Austria (BMWF-66.008/0011-II/3b/2013 and BMWFW-66.008/0011-WF/V/3b/2014).

## Signal processing and data analysis

The scoring routines require individual data vectors (text file) for the EEG and EMG recordings. The scoring routine itself consists of three individual, although complementary parts, the ARTIFACT DETECTION, the semi-automated SLEEP SCORING and the manual RESCORING that can be selected via the graphical user interface (GUI). Within the user interface, the following parameters can be set:

Sampling rate: The sampling rate of the EEG and EMG signals in Hz.

Filter order: Filter order of the Butterworth band pass applied to the EEG.

Filter settings: high and low cutoff frequencies are preset to 0.5–31.75 Hz [3] but can be adjusted if necessary.

Block length: Length of the EEG episodes to be processed in seconds (see below for root mean square calculation)

File tag: Individual markers to individualize the generated artifact, autoscoring and rescoring vectors.

## Scoring process

A complete scoring procedure starts with the ARTIFACT DETECTION and continues with the semi-automated SLEEP SCORING, followed by the manual RESCORING routine. Data inputs and outputs for each routine are specified in Table 1

**Fig. 1:**
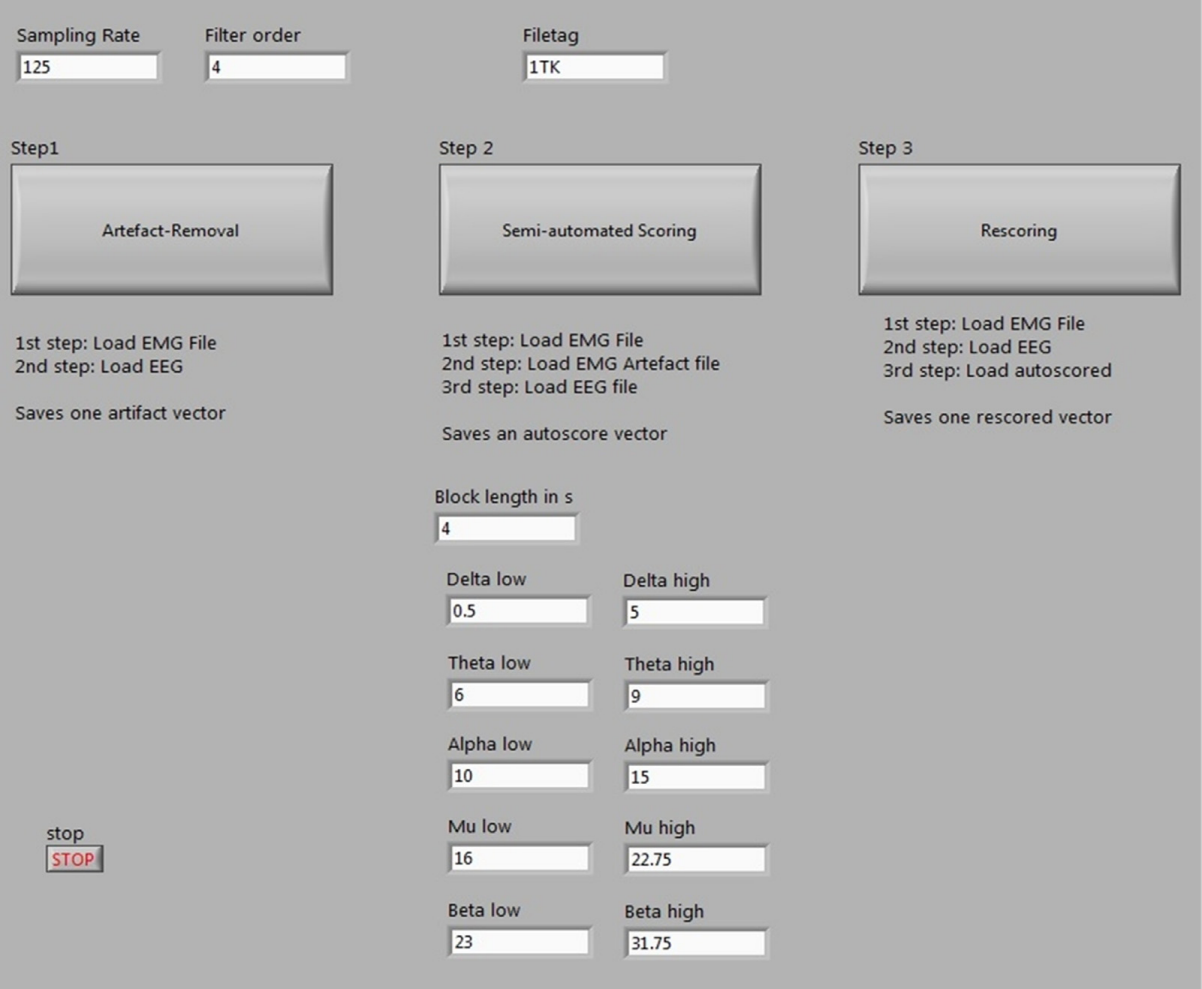
Screenshot of the Scoring-Routine GUI. The user can adjust the general settings and start the artifact, scoring or rescoring programs.

In order to start a scoring process, the user opens the “Scoring-Routine” GUI. There, the user can set sampling rate, block length and alter filter settings if necessary. The artifact detection, sleep scoring and rescoring routine can be started from the GUI by clicking the corresponding button. Fig. 1 shows a screenshot of the “Scoring-Routine”.

### ARTIFACT DETECTION (semi-automated)

To eliminate EEG/EMG artifacts, first the EMG data vector and then the EEG data vector must be selected and fed into the ARTIFACT DETECTION routine. This routine eliminates short artifacts as well as extended artifacts, frequently caused by heavy movements of the animal. Short artifacts can be eliminated by manually setting individual maximum and minimum graphical thresholds in the EMG and EEG vector plots. All amplitudes above the maximum threshold and all amplitudes below the minimum threshold will automatically be defined as an artifact.

**Fig. 2:**
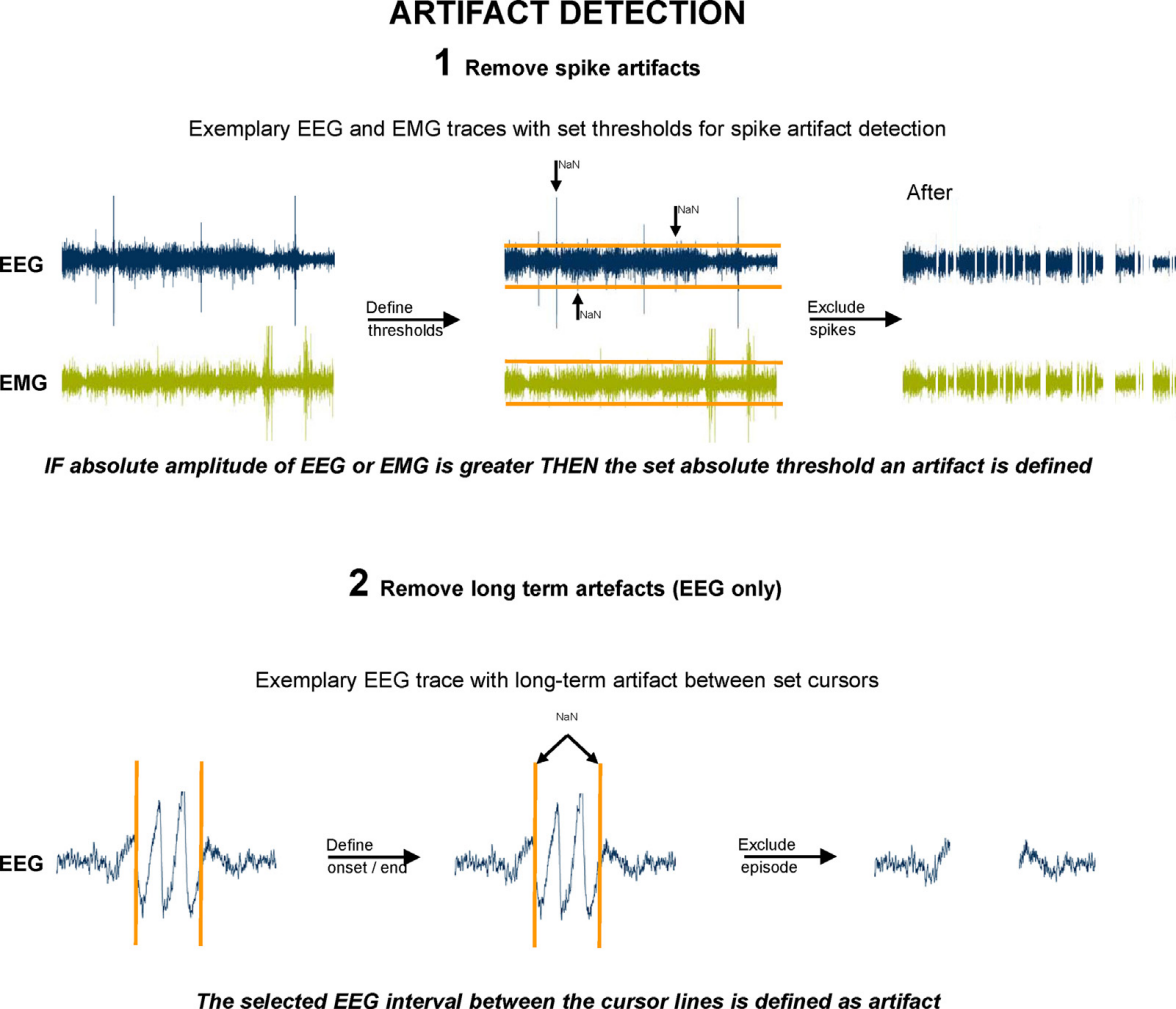
The user can define short (top) and long-term artifacts (bottom) with the ARTIFACT DETECTION routine. (1) For the removal of spike-like (short) artifacts, the user can set upper and lower amplitude threshold cursors. All data points outside these cursors are set to NaN and hence excluded from the sleep scoring analysis. (2) In order to remove long term artifacts, the user defines the onset and end of such an artifact by zooming into the EEG or EMG trace. In line with step a, all visible data points are set to NaN.

**Fig. 3:**
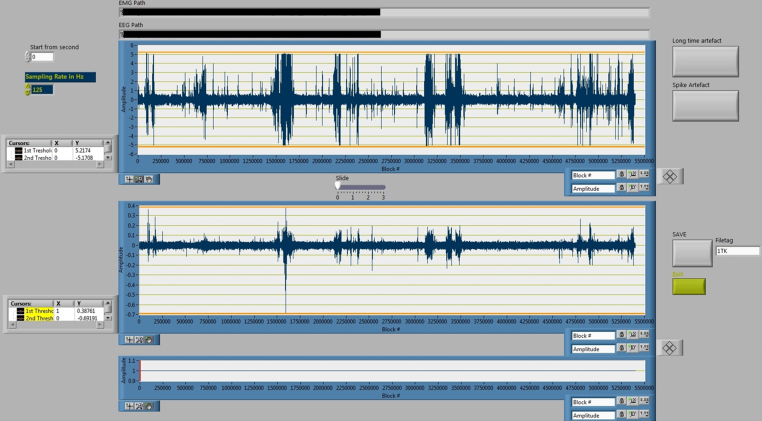
Screenshot of the ARTIFACT DETECTION GUI. For spike removal, the user can set the orange horizontal cursors as upper and lower threshold for artifact detection. A click on “Spike Artefact” removes all spikes defined by the threshold as shown in Fig. 2. The user can further zoom into the EEG or EMG trace. A click on “Long time artifact” sets all visible data points to NaN.

For the elimination of extended artifacts the user can select the affected segment of the EEG data vector. By clicking the “Long-term” artifact button the entire EEG segment displayed is defined as artifact. All “artifact” episodes are excluded from the data vectors for further analysis, labeled with NaN. A visualized scheme of the artifact detection process is shown in Fig. 2. Fig. 3 shows a screenshot of the artifact detection GUI.

### SLEEP SCORING (semi-automated)

The semi-automated sleep scoring is based on individual thresholds to be set for processed EEG and EMG recordings after loading the EMG vector (_rms), the artifact vector (_artifact) and the EEG vector. After loading the vectors, the routine automatically performs the following processing steps:

1st step: EEG filtering to the 0.5–31.75 Hz frequency band by a 3rd order Butterworth filter before it is plotted in the active “EEG window” (parameter 1) of the SLEEP SCORING GUI.

2nd step: Additional EEG filtering to particular frequency bands according to the articles of Louis et al. and [6] Fenzl et al. [3]: (*δ*: 0.5–5 Hz; *θ*: 6–9 Hz; *α*: 10–15 Hz; *μ*: 16–22.75 Hz and *β*: 23–31.75 Hz).

3rd step: Calculation of the root mean squares (RMS) for each of the above mentioned filtered signals (RMS; δ_RMS_, θ_RMS_, α_RMS_, μ_RMS_, and β_RMS_) and for the raw EMG vector (EMG_RMS_) from strictly non-overlapping episodes. The user can define the episode length with the “*Block length in s*” button of the SLEEP SCORING GUI. RMS calculation is according to following formula:

$$RMS=\sqrt{\frac{1}{N}\sum_{i=0}^{N-1}{\left|x_{i}\right|}^{2}}$$

*N* is the number of data points in any given episode and $x_{i}=[x_{1},...,x_{N}]$ represents the discrete amplitude values in a given episode.

4th step: SLEEP SCORING creates additional “DELTA and THETA graphs” (parameter 2 and parameter 3), calculated on the following equations [6], [3]:

$$DELTA=\frac{\delta_{RMS}\alpha_{RMS}}{\mu_{RMS}\beta_{RMS}}andTHETA=\frac{\theta_{{RMS}^{2}}}{\delta_{{RMS}^{\alpha }RMS}}$$

The EMG_RMS_ is the fourth parameter used for sleep analysis by plotting the function exp^(EMGRMS)^ in the “EMG activity” window within the GUI, locked to the corresponding episodes of the EEG window.

**Fig. 4:**
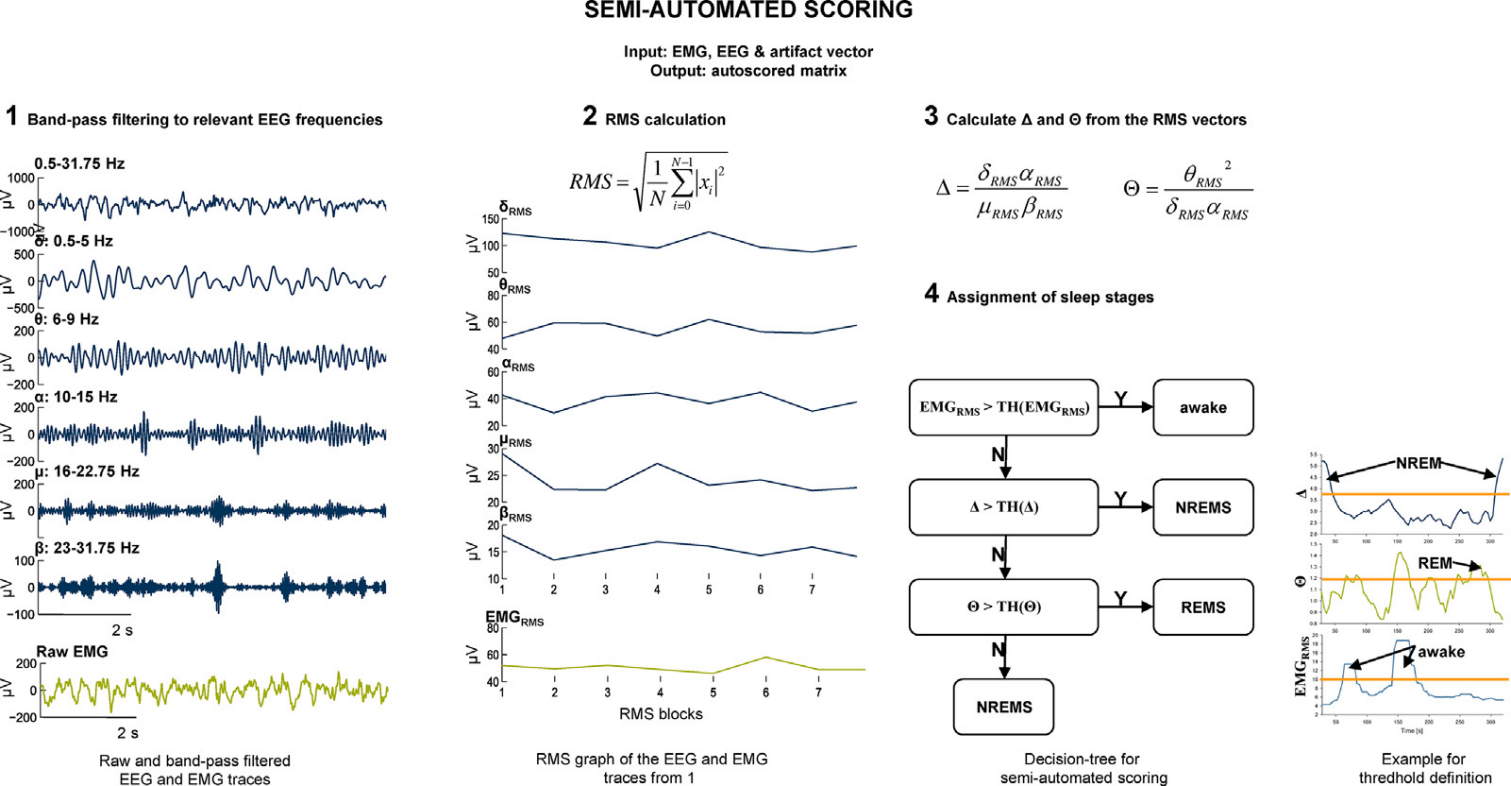
In the (semi-) automated SLEEP SCORING routine following steps are performed: (1) The EEG is filtered to different frequency bands. (2) For each frequency band and the EMG data vector the RMS for non-overlapping episodes of the block length defined in the GUI is calculated. (3) EEG parameters DELTA and THETA are calculated according to the corresponding equations. (4) Decision tree for sleep stage assignments including an example for the manually set thresholds.

**Fig. 5:**
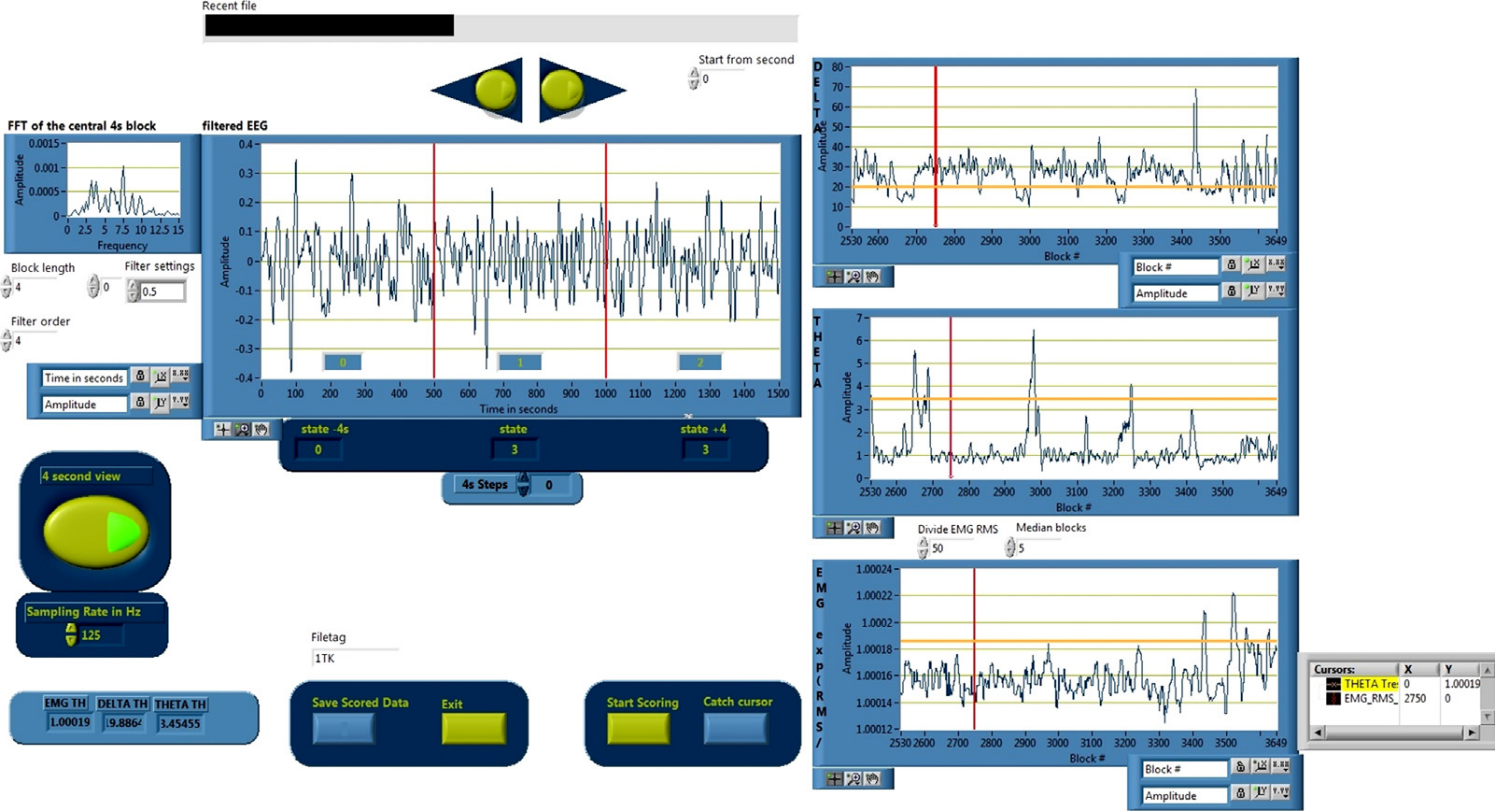
Screenshot of the semi-automated SLEEP SCORING GUI. The three graphs on the right side show the RMS parameters that are used for the decision algorithm. The user can manually set the (orange) thresholds. A click on “start scoring” starts the scoring algorithm. This procedure can be repeated and the scoring result is not saved until “Save Scored Data” is clicked. (For interpretation of the references to color in this figure legend, the reader is referred to the web version of this article.)

In each of these windows (DELTA, THETA and EMG_RMS_) the user can set a horizontal threshold cursor. These thresholds trigger a decision tree, published by Louis et al. [6], to define the sleep stage for the corresponding EEG episode. Fig. 4 shows the individual steps of EEG and EMG band pass filtering, RMS calculation and assignments of sleep stages based on the decision tree. Fig. 5 displays a screenshot of the GUI for semi-automated sleep scoring

Decision 1(performed by the user): Vigilance state AWAKE vs. SLEEP (Decision is depending on the EMG_RMS_ threshold). All episodes within the “EMG activity” window with exp^(EMGRMS)^ values above the user-defined threshold are automatically indicated as AWAKE. All episodes with exp^(EMGRMS)^ values below the user-defined threshold are automatically indicated as NOT AWAKE (low or no EMG activity equals sleep; quiet wakefulness, also indicated by low or no EMG activity may be evaluated within the RESCORING routine manually). For all sub-threshold exp^(EMGRMS)^ episodes the sleep-state NREMS or REMS has to be defined as follows.

Decision 2 (performed by the user): NREMS vs. REMS (Decision is depending on the DELTA and THETA thresholds). All episodes indicated as NOT AWAKE by decision 1 are subject to further evaluation by applying individual thresholds for DELTA and THETA assigning REMS or NREMS.

Episodes are defined as REMS when the corresponding DELTA function is below its manually set threshold while the synchronized THETA function is above its manually set threshold. All other episodes, which have not been assigned to AWAKE or REMS are defined as NREMS automatically.

The autoscored matrix consists of four columns:

1st column: (semi-) autoscored behavioral states, assigned to each individual epoch (AWAKE, NREMS, REMS)

2nd column: DELTA values

3rd column: THETA values

4th column: EMG_RMS_ values

### RESCORING (manually)

**Fig. 6:**
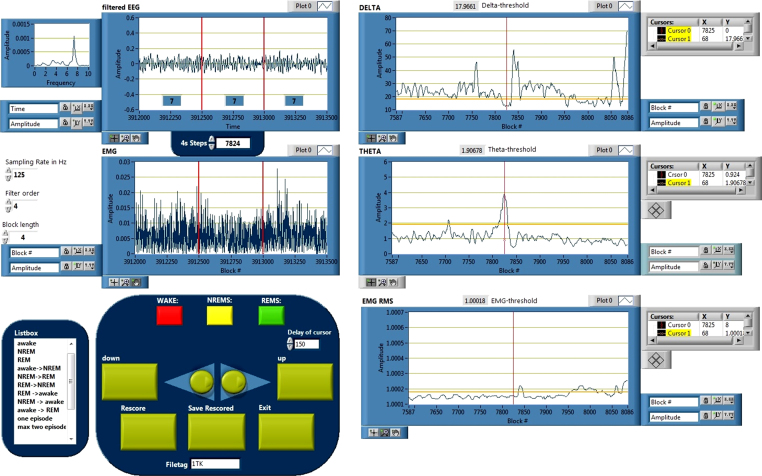
Screenshot of the RESCORING GUI. The (semi-) automated scoring vector can be manually corrected with this routine. Individual vigilance states and state transitions can be selected using the listbox on the left. The central section in the “filtered EEG” and “EMG” windows can be re-evaluated by clicking on the “WAKE”, “NREMS” or “REMS” button. A click on “Save Rescored” saves the changes applied.

This routine provides the opportunity to manually select and rescore/correct each episode individually by loading the (semi-) autoscored matrix in the RESCORING routine together with the raw EMG and EEG files. From a “list box” menu all previously assigned vigilance states and/or all possible state transitions can be selected and plotted for re-evaluation. For the selected epochs, the EMG and EEG raw signal is displayed and the corresponding DELTA, THETA and EMG_RMS_ are marked with a cursor. The user can change each (semi-) autoscored behavioral state (AWAKE, NREMS, REMS) in the GUI. Re-evaluated behavioral states are assigned to each individual epoch (AWAKE, NREMS, REMS). Fig. 6 presents the GUI of the rescoring routine.

## Additional information

The sleep scoring routine represents a straight forward and fast approach to detect the behavioral states AWAKE, NREMS and REMS, based on EEG and EMG recordings from mice. The user can also score sleep behavior of rats and other laboratory animals after adaption of the selected frequency bands and its corresponding equations that underlie the DELTA and THETA calculation. The sleep scoring routines are especially designed for laboratories whose research focus is not mainly sleep research, although the tools can easily be adapted and extended to a more in-depth analysis as required for evaluation of state transitions, state durations or Fast Fourier analysis of particular frequency bands (e.g., slow wave activity). By keeping the presented tool straightforward, basic sleep analysis can now be included as an additional readout to complex behavioral, circadian dependent questions related to chronopharmacology, chronotoxicology, chronobiology, learning and memory etc.

We performed a random sample test to evaluate the performance of our sleep scoring tool. Two very experienced scorers semi-automatically and independently analyzed recordings of 14 animals from our laboratory data pool and manually rescored five of these (semi-) automatically generated data files.

The agreement between the two scorers’ semi-automatic scoring results was 90.8% in average (*n *= 14 min: 84.8%; max: 95.5%) and agreement after sample rescoring was 91.1% (*n *= 5; min: 85.9%; max: 95.4%). Cohen’s Kappa was 0.850 (CI: 0.847–0.851) for the autoscored data as well as 0.851 (0.847–0.854) for the rescored data. Results indicate excellent (independent) agreement between both scorers.

The fraction of rescored episodes was 5.0% (min: 2.1%; max: 9.0%).

The agreement of autoscoring versus manual rescoring was 95.0% (SEM: 0.67%) which is well in the range of other automated sleep scoring approaches described in literature as presented in Table 2.

The LabVIEW routine as well as a “quick start” manual can be obtained from the authors. The LabVIEW Runtime engine is freely available from the provider (National Instruments, Austin, TX, USA, http://www.ni.com/support/).

## Figures and Tables

**Table 1:** Required inputs and created outputs for the scoring routines.

Routine	Input	Output
ARTIFACT DETECTION	(1) EMG data vector (raw data file) (2) EEG data vector (raw data file)	Artifact vector *file annex: _artifact*

SLEEP SCORING	(1) EMG data vector (_rms/ RMS- filtered EMG data vector) (2) Artifact vector (3) EEG data vector (raw data file)	Autoscored matrix *file annex: _autoscored*

RESCORING	(1) EMG data vector (raw data file) (2) EEG data vector (raw data file) (3) Autoscored matrix	Rescored vector *file annex: _rescored*

**Table 2:** Rate of agreement between automated and manual sleep scoring represented in literature.

Author	Agreement	Animal	
	auto- vs. manual scoring	Inter-rater	
Rytkönen [8]	93%	88–92%	Rat mouse
Stephenson [9]	87-92%		Rat
Gross [5]	80%	83%	Rat
Louis [6]	88%	88%	Rat
Costa-Miserachs [2]	94%	96%	Rat
Benington [1]	88–91%	95%	Rat
